# Reserve-building activities in multiple sclerosis patients and healthy controls: a descriptive study

**DOI:** 10.1186/s12883-015-0395-0

**Published:** 2015-08-12

**Authors:** Carolyn E. Schwartz, Armon Ayandeh, Murali Ramanathan, Ralph Benedict, Michael G. Dwyer, Bianca Weinstock-Guttman, Robert Zivadinov

**Affiliations:** DeltaQuest Foundation, Inc., 31 Mitchell Road, Concord, MA 01742 USA; Departments of Medicine and Orthopaedic Surgery, Tufts University Medical School, Boston, MA USA; Department of Pharmaceutical Sciences, School of Medicine and Biomedical Sciences, State University of New York, Buffalo, NY USA; Department of Neurology, School of Medicine and Biomedical Sciences, University of Buffalo, State University of New York, Buffalo, NY USA; Buffalo Neuroimaging Analysis Center, Department of Neurology, School of Medicine and Biomedical Sciences, State University of New York, Buffalo, NY USA; Department of Biomedical Informatics, University of Buffalo, State University of New York, Buffalo, NY USA; MR Imaging Clinical Translational Research Center, School of Medicine and Biomedical Sciences, University at Buffalo, State University of New York, Buffalo, NY USA

## Abstract

**Background:**

Cognitive reserve has been implicated as a possible protective factor in multiple sclerosis (MS) but to date no study has compared reserve-building activities across disease course or to healthy controls. This study aims to describe differences in reserve-building activities across the MS disease course and healthy controls.

**Methods:**

Secondary analysis of a cross-sectional cohort study that included 276 healthy controls, and subjects with clinically isolated syndrome (CIS; n = 67), relapsing-remitting MS (RRMS; n = 358) and secondary progressive MS (PMS; n = 109). Past reserve-building activities were operationalized as occupational attainment and education. Current activities comprised 6 strenuous and 6 non-strenuous activities, including 5 reserve-building activities and television-watching. Multivariate Analysis of Variance models examined group differences in past and current activities, after adjusting for covariates.

**Results:**

There were group differences in past and current reserve-building activities. SPMS patients had lower past reserve-building activities than healthy controls. All forms of MS engaged in fewer strenuous current reserve-building pursuits than healthy controls. RRMS read less than healthy controls. SPMS engaged in fewer job-related non-strenuous activities. All MS groups watched more television than healthy controls.

**Conclusions:**

MS patients show significantly fewer past and present reserve-building activities. Although it is difficult to establish causality without future prospective studies, lifestyle-modifying interventions should prioritize expanding MS patients’ repertoire of strenuous and non-strenuous activities.

## Background

The concept of resilience has been the focus of study via diverse social scientific disciplines, including behavioral medicine [1], health psychology [2], epidemiology [3], and education research [2]. Recent clinical research in neurology has revealed that cognitive reserve – a property of the nervous system enhanced by past and current salutogenic stimulating activities -- is associated with better cognitive functioning in the face of neurologic illness or injury [4]. Recent work has documented that past and current stimulating activities may be protective against progression in a broad range of disability domains in multiple sclerosis (MS) [5]. The multi-dimensionality of the factors documented to contribute to resilience is notable, going beyond cognitive activities or outcomes and extending into physical, creative, intellectual, spiritual, and cultural enrichment. Consequently, we believe the nomenclature should be changed to broaden the implied dimensionality of reserve by referring to the concept of *reserve* rather than “cognitive reserve”.

Reserve is conceptualized as arising from inborn, past, and current resources, and has been operationalized by measurable indicators. Inborn reserve or *“brain reserve*” has been operationalized as intracranial volume [6], head circumference [6], measured intelligence quotient in early life, and genetic/environmental modifiers [7]. *Past reserve-building activities* derive from past enrichment and achievement, and have been measured as educational and occupational attainment as well as childhood exposure to stimulating cultural and educational pursuits [8, 9]. *Current reserve-building activities* refer to current enrichment pursuits, and have been measured as current cultural, intellectual, physical, and spiritual leisure activities [4, 9]. These reserve-building pursuits may require new learning, leading to the development of more dendrites, dendritic spines, synapses, and perhaps even cells, all of which contribute to reserve. In particular, diverse current reserve-building pursuits may be important to maintain reserve by ensuring that more areas of the brain and interconnections remain active and fit. The concept of reserve provides a parsimonious and inclusive framework for examining how an individual can enhance health and well-being by current pursuits that build on childhood experiences and innate capacity [10].

The growing evidence base supporting the relevance and importance of reserve has generally focused on its impact in people dealing with neurological illness or injury, including MS [11], brain injury [12], Parkinson’s disease [13], Alzheimer’s disease [14], cancer chemotherapy [15], and lead exposure [16]. To our knowledge, no work has been done examining multidimensional indicators of reserve in healthy individuals and comparing them to people with an illness. Although it is common practice to compare patients to healthy controls on the basis of cognitive or neuropsychiatric symptoms in studies of MS patients, it is not known how leisure pursuits that would relate to reserve differ between patients and healthy controls. Such a comparison would be useful not only for understanding normative levels of reserve; they would also be helpful for elucidating how levels differ before and after illness. We thus sought to describe indicators of past and current reserve-building activities in a secondary analysis of a relatively large cohort of people with MS and healthy controls.

## Methods

### Sample

This secondary analysis utilized data from an ongoing prospective study of clinical, genetic and environmental risk factors in MS at the MS Center of the State University of New York at Buffalo which enrolled over 1,000 subjects with clinically isolated syndrome (CIS) [2, 17, 18], MS, healthy controls, and other neurologic diseases (OND) [19, 20]. The sample included 67 (8.3 %) people with CIS; 358 (44.2 %) people with relapsing-remitting MS (RRMS) and 109 people (13.5 %) with secondary progressive MS (SPMS). There were also 276 age- and sex-matched healthy controls. The inclusion criteria for this sub-analysis were presence of sufficient questionnaire data to obtain current and past reserve-building activities variables (i.e., the respondent was not missing data on the items assessing past and current reserve-building activities). The exclusion criteria were presence of relapse and steroid treatment in the 30 days preceding study entry for CIS and MS patients, pre-existing medical conditions known to be associated with brain pathology (cerebrovascular disease, positive history of alcohol abuse) and pregnancy. Healthy controls needed to meet the health-screen requirements, and had to have a normal physical and neurological examination. They were recruited from hospital personnel, or were respondents to a local advertisement. Table 1 provides demographic and clinical characteristics of the MS patient groupings and age-, sex- and race-matched healthy controls.

**Table 1:** Study participant demographics

Variable	HC	CIS	MS RR	MS Progressive			
	N = 276	N = 67	N = 358	N = 109	Test Statistic	P-value	
Gender: % female	61.09	62.69	64.53	61.47	0.29	0.8298	
Mean Age (sd)	46.93 (15.76)	39.45 (10.88)	44.16 (10.71)	53.91 (8.69)	23.89	<0.0001	**
Mean Age at Diagnosis (sd)	N/A	36.31 (10.78)	34.82 (9.39)	36.21 (10.09)	1.22	0.2953	
Mean EDSS (IQR)	N/A	1.50 (1–2)	2.00 (1.5-3)	6 (5–6.5)	235.7	<0.0001	**
Mean BMI (sd)	27.31 (5.70)	27.17 (5.86)	27.42 (5.90)	26.10 (6.01)	1.38	0.2475	
Employment Status %							
Full-time	48.22	63.08	44.89	11.32	36.33	<0.0001	***
Part-time	15.81	10.77	11.93	10.38	0.96	0.4109	
Homemaker	3.16	7.69	3.41	4.72	0.67	0.5736	
Student	7.91	4.62	1.99	0	10.62	<0.0001	***
Unemployed	8.3	6.15	7.1	5.66	0.33	0.8052	
Retired	13.83	3.08	6.53	24.53	9.69	<0.0001	***
Disabled	0	3.08	22.44	41.51	59.37	<0.0001	***
Other	2.77	1.5	1.7	1.89	0.27	0.8436	
Education							
High school not completed	1.99	1.54	3.71	7.55	1.9	0.1273	
Graduated high school	15.54	12.31	17.14	21.7	1	0.3944	
Some college/Associate/Technical Degree	34.66	38.46	36.29	33.96	0.17	0.9155	
Bachelor’s degree	25.9	30.77	23.14	19.81	1.05	0.3699	
Graduate/Post-graduate	21.91	16.92	19.71	16.98	0.54	0.6557	
Race							
White	83.59	92.31	92.05	95.28	5.03	0.0018	**
Hispanic/Latino	1.56	3.08	1.99	0.94	0.41	0.749	
Black/African-American	8.59	3.08	4.83	2.83	2.26	0.0801	
Asian	3.9	1.54	0.56	0	4.46	0.0041	**
American Indian/Alaska Native	0.39	0	0	0	1	0.3176	
Other	1.95	0	0.57	0.94	2.7	0.045	*

*HC* = Health Control; *CIS* = Clinically Isolated Syndrome; *MS RR* = MS Relapsing Remitting; The F-Statistics and P-values shown are from tests for differences between disease group for the given variable

### Procedure

All subjects were assessed with a structured questionnaire administered in-person by a trained interviewer unaware of the subjects’ disease status [2]. This study was approved by the State University of New York at Buffalo Institutional Review Board (HSIRB #NEU2490109A) and written informed consent was obtained from all subjects.

### Measures

The questionnaire contained information related to demographic characteristics, presence of autoimmune and other concomitant diseases, vascular risk factors and environmental factors, as well as information about habits. There were a set of questions addressing physically strenuous (i.e., exercise) activities as well as non-strenuous activities (e.g., hobbies or other pastimes). These questions reflected activities similar to those included in questionnaires investigating reserve-building activities [9, 13]. This analysis utilized items in the questionnaire containing information on activities relevant to building reserve and covered a subset of relevant past and current leisure activities. Based on the psychometric analyses described below, we created derived measures of reserve-building activities.

### Statistical analysis

#### Data reduction

A series of data-reduction steps were used to generate composite scores for past and current reserve-building activity scores for analyses.

A past reserve-building activity score was created as educational and occupational attainment scores. An occupational attainment score was created using the O*NET OnLine Job Zones (ranging from one to five) based on how much education people need to do their work, how much related experience people need to do their work, and how much on-the-job training people need to do their work (http://www.onetonline.org/help/online/zones). When the job entry was not sufficiently specific to match only one O*NET job, the Job Zones for the relevant jobs were averaged. For example, a social worker may work with children, families, and schools (job zone 4), or with persons with mental health and substance abuse issues (job zone 5), giving them an occupational attainment score of 4.5. It should be noted that the focus of the O*NET coding was on level of occupational attainment of most recent/current job, not current employment status.

Current reserve-building activities were divided into strenuous and non-strenuous activities. Examples of strenuous activities included contact sports, aerobics, swimming, and wrestling. Example of non-strenuous activities included reading, browsing the internet, job related e-mail, meditation, and doing puzzles. Television-watching was also tracked in the data set and included in the analysis; it was considered a pastime unlikely to build reserve and which competed with reserve-building activities.

Survey questions addressing each activity were respectively analyzed using exploratory factor analysis (EFA) in Mplus statistical software [21]. An Mplus EFA was run on 18 strenuous items, leading to the eventual dropping items of that did not load above 0.40 on any of the factors (i.e., tai chi, other physical activity, and other aerobics). Based on the factor loadings and exercise-science conventions, we grouped the different strenuous activities into the following scores: *Job related* (job-related walking and moving); *Organized Sports* (contact sports, court sports, field sports); *High Impact* (aerobics, cross-training, running, weights); *Low Impact* (swimming, yoga, walking); *Fighting Sports* (wrestling, boxing); *Computer/television-Related Exercise*. An average score was calculated for each of the strenuous factors because the average was considered to be more interpretable than a sum. Each factor score was the average of the items it represented. A Strenuous Activity Summary Score was created by summing the strenuous factor scores.

An Mplus EFA with between one and nine factors was run on the 14 non-strenuous items using the ULS estimator and quartimin rotation. The output suggested a six- factor model based on the Kaiser criterion (Eigenvalues > 1). The quartimin rotated loadings were then used to group variables together, resulting in a *reading* factor (reading newspapers and reading magazines), *television* factor (television only), *internet* factor (browsing, social networking), *job-related* factor (job related reading, e-mails), *spiritual* factor (prayer, meditation), and *game* factor (cards, video games and puzzles). Each factor score was the average of the items it represented. A Non-Strenuous Activity Summary Score was created by summing the non-strenuous factor scores.

#### Multivariable analysis

To examine group differences in levels of past and current reserve-building activities, we tested three sets of multivariate analysis of variance (MANOVA) models with a Type I error rate of 5 %. MANOVA provides an omnibus test of statistical significance. If this omnibus test was statistically significant for the key independent variable (i.e., patient group), then post-hoc comparisons were run to identify which groups were statistically different from healthy controls. This statistical approach allows for control over the inflation of Type I error rate due to multiple comparisons [2]. A further strategy for adjusting for multiple comparisons was to prioritize the consistency of the four MANOVA output statistics (i.e., Wilk’s lambda, Lawley-Hotelling trace, Pillai’s trace, and Roy’s largest root). Group differences were interpreted when all four statistics were statistically significant [2]. A more conservative interpretation was used when between one and four of the MANOVA statistics was significant.

The dependent variables were: (a) past reserve-building activitiy scores; (b) strenuous current activity summary scores; (c) non-strenuous current activity summary scores. The independent variable was disease group (CIS, RRMS, and SPMS, with healthy controls as the comparison/referent group); age and gender were covariates. The MANOVA analyses of current Strenuous and Non-Strenuous activities also adjusted for education and occupational attainment to control for the effects of past reserve-building activities in explaining current reserve-building activities. Post-hoc estimation was done using multivariate regression. The MANOVA and regression analyses were implemented using Stata 13 [22].

## Results

### Past reserve-building activities

The MANOVA results suggested that there were possible group differences in past reserve-building activities, after adjusting for age and gender. The four MANOVA summary statistics were, however, not consistent (Table 2). Roy’s largest root was significant (i.e., p < 0.01) but Wilk’s lambda, Pillai’s trace, and Lawley-Hotelling trace were not significant. Post-hoc tests suggested that SPMS patients had lower occupational attainment and education level than healthy controls (p < 0.003 and 0.007, respectively).

**Table 2:** MANOVA test results

Source		N	Statistic	df	F(df1,	df2)	=F	Prof > F
Past Reserve-Building Pursuits Model: Disease Group	W	729	0.98	3	6	1444	1.97	<0.07
	P		0.02		6	1446	1.97	<0.07
	L		0.02		6	1442	1.98	<0.07
	R		0.02		3	723	3.94	<0.01
Strenuous Activities: Disease Group¥	W	704	0.68	3	18	1938	15.69	<0.0001
	P		0.32		18	2061	13.89	<0.0001
	L		0.46		18	2051	17.53	<0.0001
	R		0.44		6	687	50.94	<0.0001
Non-Strenuous Activities: Disease Group¥	W	702	0.92	3	18	1932.3	3.34	<0.0001
	P		0.08		18	2055	3.31	<0.0001
	L		0.09		18	2045	3.36	<0.0001
	R		0.06		6	685	7.03	<0.0001

*W* = Wilks' lambda; *P* = Pillai's trace; *L* = Lawley-Hotelling trace; *R* = Roy's largest root

¥ Adjusted for education and occupational attainment

### Current reserve-building activities

#### Strenuous activities

**Fig. 1:**
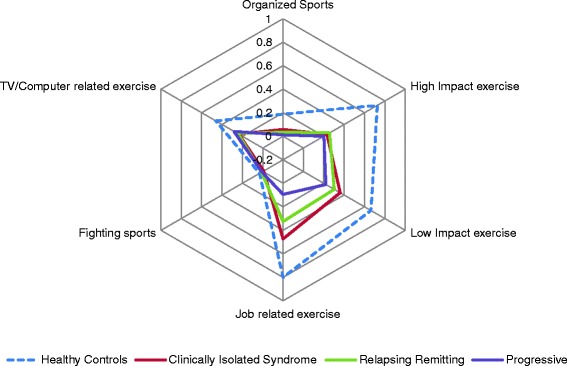
Strenuous activity scores by disease group. Healthy control scores are adjusted for age, gender, and the other disease groups are adjusted for age, gender, years since symptom onset, education, and occupational attainment

There were consistent group differences in strenuous current reserve-building pursuits (p < 0.0001), after adjusting for age, gender, education, and occupational attainment (Table 2; Fig. 1). Compared to healthy controls, people with all forms of MS engaged in fewer high-impact exercise (all p < 0.0001), low-impact exercise (all p < 0.0001), organized sports (all p < 0.0001), and job-related strenuous active pursuits (all p < 0.0001) (Table 3). RRMS and SPMS engaged in less computer- or television-based exercise (p < 0.02 and 0.05, respectively), and SPMS did fewer fighting sports (p < 0.05) (Table 3).

**Table 3:** Summary of Post-Hoc Comparisons

	Disease Group			
Dependent Variable	Healthy Control (referent)	CIS	RRMS	PMS
Past Reserve-Building Pursuits				
Education	\			-
Occupational Attainment	\			-
Current Reserve-Building Pursuits:				
Strenuous				
High impact	\	-	-	-
Low impact	\	-	-	-
Fighting Sports	\			-
Organized sports	\	-	-	-
Job-related exercise	\	-	-	-
TV/computer-related exercise	\		-	-
Current Reserve-Building Pursuits:				
Non-Strenuous				
Reading	\		-	
Internet usage	\			
Job-related	\			-
Games	\			+
Spiritual	\			
TV	\	+	+	+

¥ Adjusted strenuous and non-strenuous for Education and Occupational Attainment

Symbols in the columns reflect the direction of the relationships by participant group, with ‘-‘ reflecting negative association and ‘+’ reflecting positive association. Slash (‘\’) reflects that this was the referent group to which all other groups were compared

#### Non-strenuous activities

**Fig. 2:**
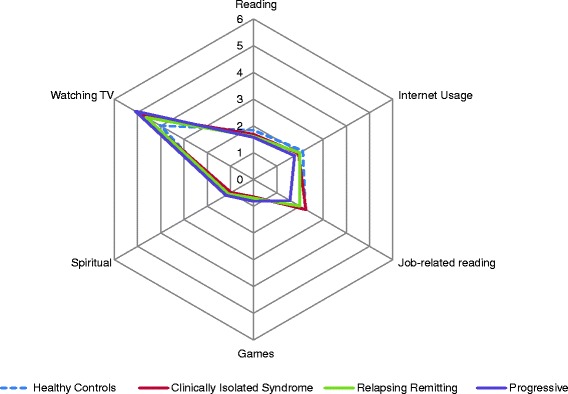
Non-strenuous activity scores by disease group. Healthy control scores are adjusted for age, gender, and the other disease groups are adjusted for age, gender, years since symptom onset, education, and occupational attainment

There were consistent group differences in non-strenuous reserve-building pursuits (p < 0.0001), after adjusting for age, gender, education, and occupational attainment (Tables 2 and 3; Fig. 2). RRMS read less than healthy controls (p < 0.01). SPMS engaged in less job-related reading and internet usage (p < 0.0001) and played more games (p < 0.05). CIS, RRMS, and SPMS watched more television than healthy controls (p < 0.02, 0.001, and 0.0001, respectively), but were similar in regard to internet usage, and spiritual pursuits.

## Discussion

Although past research has documented a protective effect of current reserve-building activities on MS disability progression [5], our findings suggest that people with all forms of MS engaged in substantially fewer of the measured strenuous and non-strenuous current reserve-building pursuits than healthy controls. Further, they spent more time watching television, a leisure time activity that would not be considered stimulating or likely to maintain complex neural pathways. Since there is limited time in each day, time spent watching television necessarily takes away from available time for other leisure pursuits that may be health-enhancing. Accordingly, lifestyle-modifying interventions should prioritize replacing television-watching with more stimulating activities.

Our finding of a possible trend for people with progressive MS to report lower educational and occupational attainment has implications worth considering. First, it is possible that the illness itself may have limited achievement, since MS often strikes during young adulthood. Second, these putative differences may reflect that even education and occupation are relevant to current reserve-building activities; for example, it is possible that lower levels of past reserve-building activities pre-dispose individuals to lower levels of current reserve-building activities. Since a person builds his/her career over time, if s/he has MS, s/he will be more likely to be out of the work force earlier than expected. This may impact occupational attainment, as well as education level if continuing (i.e., graduate) education is at play. Again, these results should be interpreted with caution because the metric suggesting this statistical difference -- Roy’s largest root – may be more prone to significance than the other metrics [23].

Our findings have implications for understanding how MS affects people in ways beyond symptom experience, and suggest possible paths for intervention. While current reserve-building pursuits have been shown to have a protective effect on MS disease progression [5], our data suggest that MS patients participate in substantially fewer current strenuous reserve-building pursuits than healthy controls (Table 3). These findings are consistent with recent documentation that 80 % of people with MS do not meet recommended levels of moderate-to-vigorous physical activity [24, 25]. It is possible that early MS symptoms lead people to drop strenuous pursuits as they focus their energies on maintaining priority roles, such as employment and home-management activities. Motl recommends a paradigm shift in how exercise training is promoted within MS care, to focus on ‘lifestyle physical activity’ rather than ‘exercise training for fitness’ [26].

Our findings also suggest that other stimulating activities that are non-strenuous are also less prevalent among people with MS than among healthy controls. While some of these activities are linked to employment (i.e., job-related reading and email), others are not (e.g., reading, spiritual). Recent work done by members of our research team has documented that activities such as participating in a group or organization, traveling, visiting museums, attending lectures, participating in arts and crafts, cooking as a hobby, etc., are all associated with lower symptom burden cross-sectionally, and with slower disease progression over time. Even severely disabled people with MS were able to attain high scores on a reliable and content-valid measure of reserve, supporting the idea that these protective pursuits are accessible across the disability spectrum.

A logical next step in this line of research involves early intervention with people with MS. Focusing on promoting both strenuous and non-strenuous current reserve-building pursuits, such work would require helping patients to identify activities they would like to do across a range of domains, and helping them to mitigate perceived barriers and ensure regular practice of such pursuits. Similar to the paradigm shift proposed by Motl [26], such intervention work would need to help individuals expand their concept of salutogenic practice, and integrate such into their daily lives. An important first step might be to disable their television!

While our findings are interesting and generate hypotheses and intervention work for future research, the limitations of the present work must be acknowledged. First, as with any post-hoc study analysis, our data were not collected for our stated objectives; accordingly the operationalization of reserve was not as robust and multi-dimensional as it would be if we used the current validated measure of the construct [9, 27]. Since we did careful psychometric analysis of the items included in the reserve operationalization used in this study, we believe that it is a relatively valid proxy for reserve. Second, the cross-sectional nature of the data limits causal inference, such as these descriptive differences merely reflecting the disease itself. Past research done by members of our group has, however, suggested that even severely disabled MS patients can engage in current reserve-building pursuits that lead to a similar score as less disabled patients [5]. Nonetheless, future work might build on this study by comparing healthy controls and MS patient groups *over time* using the validated measure of reserve [9] to allow for causal inference.

## Conclusions

In summary, our study documented that people with MS engage in fewer strenuous and non-strenuous current reserve-building pursuits, while watching more television than healthy controls. We believe the implications of these findings provide clear pathways for improving the health and well-being for people with MS, including lifestyle interventions that expand their repertoire of strenuous and non-strenuous reserve-builiding activities.

## Abbreviations

CIS: Clinically isolated syndrome
MS: Multiple sclerosis
RRMS: Relapsing-remitting multiple sclerosis
SPMS: Secondary progressive multiple sclerosis
